# Idiopathic ventricular fibrillation with repetitive activity inducible within the distal Purkinje system

**DOI:** 10.1016/j.hrthm.2019.04.012

**Published:** 2019-08

**Authors:** Michel Haissaguerre, Ghassen Cheniti, William Escande, Alexandre Zhao, Mélèze Hocini, Olivier Bernus

**Affiliations:** ∗ IHU Liryc, Electrophysiology and Heart Modeling Institute, Foundation Bordeaux Université, Bordeaux, France; † Université de Bordeaux, Centre de Recherche Cardio-Thoracique de Bordeaux, INSERM U1045, Bordeaux, France; ‡ Bordeaux University Hospital (CHU), Cardiac Electrophysiology and Cardiac Stimulation Team, Bordeaux, France

**Keywords:** Premature beat, Purkinje system, Reentry, Sudden death, Ventricular fibrillation

## Introduction

Ventricular ectopy mostly originating from the distal Purkinje system is observed in up to 30% of cases of idiopathic ventricular fibrillation (VF).^1^, ^2^, ^3^, ^4^, ^5^ These ectopies commonly have short coupling R-on-T intervals. However, the Purkinje triggers are often transient, present only during short arrhythmic periods, which prevents recognition of this condition and its treatment. Here, we report 2 patients with idiopathic VF in whom altered electrophysiological properties and inducible repetitive activity were demonstrated in the peripheral Purkinje system in the absence of clinical short-coupled ectopy.

## Case reports

### Case 1

A 46-year-old woman with no family history of sudden cardiac death was admitted after an aborted episode of VF. She collapsed in her kitchen and lost consciousness. Her husband called emergency medical services immediately and started basic cardiopulmonary resuscitation with chest compressions. Emergency medical services arrived 18 minutes later and found that the patient had VF, which was successfully converted to sinus rhythm using an external defibrillator.

**Figure 1:**
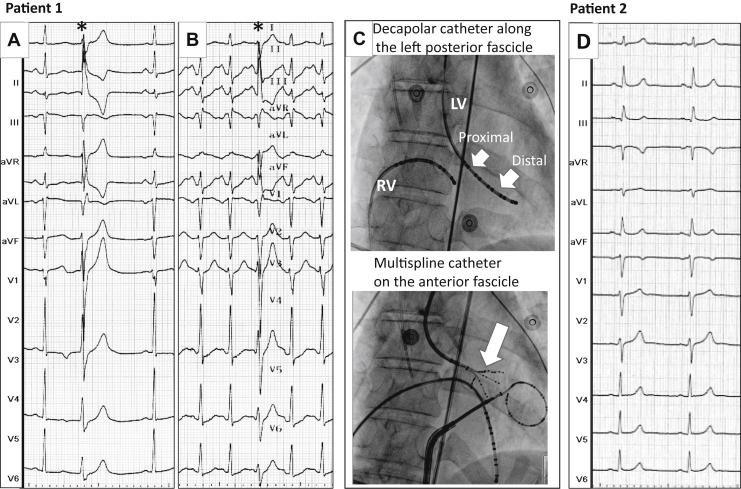
**A:** Patient 1. Twelve-lead electrocardiogram recorded in the intensive care unit after resuscitation. Fascicular ectopy with a long coupling interval is shown by the *asterisk.***B:** Isoproterenol test shows identical rare fascicular ectopy without a short coupling interval. **C:** Fluoroscopic anterior views showing the position of catheters during mapping of the Purkinje system: a decapolar catheter along the posterior fascicle (**top***arrows*) and a multispline 5-spline and 20-pole catheter on the anterior fascicle (**bottom***arrow*). The circular mapping catheter is positioned in the pericardium. Catheter recordings are shown in Figure 2. **D:** Patient 2. Twelve-lead electrocardiogram recorded during sinus rhythm. LV = left ventricle; RV = right ventricle.

On admission to the intensive care unit, the patient’s electrocardiogram (ECG) showed sinus rhythm with rare premature ventricular beats, displaying a narrow QRS duration and long coupling interval (Figure 1A). This pattern was suggestive of a Purkinje fascicular origin. She recovered normal brain function within 1 week. Cardiac evaluation including cardiac catheterization, coronary arteriography, and delayed enhancement magnetic resonance imaging demonstrated no structural heart disease. Serial ECGs at rest showed QRS duration of 84 ms and QT/QTc values of 400/422 ms. Result of exercise stress test was normal. Pharmacologic tests performed using isoproterenol (Figure 1B), ajmaline, and adrenaline did not induce ventricular premature beats and showed no evidence of catecholaminergic ventricular tachycardia (VT), Brugada syndrome, or long QT syndrome, respectively, thus leading to the diagnosis of idiopathic VF.^2^, ^3^, ^4^, ^5^

**Figure 2:**
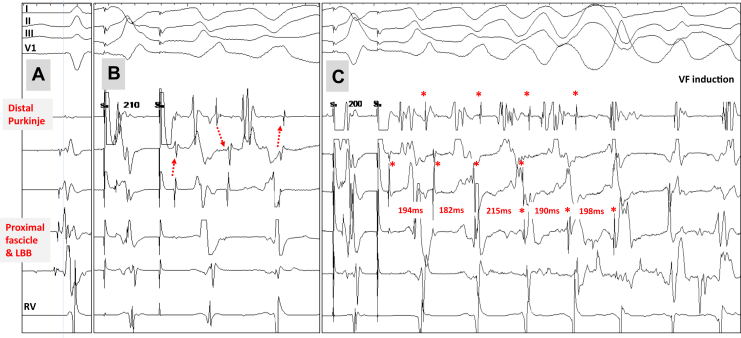
Patient 1. **A:** Recordings during sinus rhythm with the decapolar catheter along the proximal left bundle branch and posterior fascicle (2 bipoles) and distal Purkinje system (3 bipoles) as shown in Figure 1C. The same catheter positioning was kept during programmed stimulation performed at the most distal site (“S” in panels B and C). **B:** Complex response during S2 extrastimulation (drive cycle 400 ms) from the distal Purkinje at a coupling interval of 210 ms is shown. Note that only the peripheral Purkinje system is activated (no activity in proximal fascicle) and shows beat-to-beat changes in activation sequence *(red arrows).***C:** Extrastimulation from the distal Purkinje at a coupling interval of 200 ms. A Purkinje potential *(red asterisks)* precedes each QRS complex with beat-to-beat changes in activation sequence in the distal Purkinje *(red asterisks)* and then the proximal fascicle for 3 beats *(red asterisks).* The *red* values indicate the cycle lengths. Dissociated activity is observed between contiguous bipoles, with the Purkinje potentials intermittently absent or present in 1 lead, “independent” of the contiguous bipoles. A polymorphic configuration of QRS complexes can be seen on the surface electrocardiogram. A sustained ventricular fibrillation (VF) is then induced, requiring electrical cardioversion. LBB = left bundle branch; RV = right ventricle.

Electrophysiological study was performed using 2 decapolar catheters positioned in the right and left ventricles. No prolonged anterograde conduction in the His-Purkinje system occurred during sinus rhythm (HV 47 ms). Programmed ventricular stimulation was performed using a maximum of 3 extrastimuli (S2, S3, S4) at 2 drive cycle lengths (600 and 400 ms) from the right ventricular apex and then from the left ventricle. No polymorphic VT or VF was inducible from the right ventricle. The left-sided decapolar catheter was placed along the posterior Purkinje fascicle, with the distal electrodes on the distal (peripheral) Purkinje and the proximal electrodes on the proximal fascicle (defined as local Purkinje-to-myocardium time >20 ms) and left bundle branch (Figures 1C and 2A). Although we cannot distinguish between the left branch and the distal common His bundle, there was at least one more cm proximal in the recording catheter where His-bundle potentials could be recorded (as suggested by the low position of the catheter shown in Figure 1C). With an S2-S3 extrastimulus protocol delivered from the left distal Purkinje electrodes, repetitive ventricular beats were induced with progressive QRS morphologic changes from one beat to the next (Figure 2B). The ventricular beats were consistently associated with distal Purkinje potentials and had a variable activation sequence and cycle, although the proximal Purkinje fascicle/bundle branch potentials were slower or absent. These polymorphic VTs thus were not compatible with either bundle branch reentry using the right and left branches or the (monomorphic) types of fascicular reentry.^6^, ^7^ One episode of polymorphic VT degenerated into VF requiring electrical cardioversion (Figure 2C). The left anterior fascicle was mapped using a multispline 20-electrode catheter (Figure 1C) with similar findings, including induction of VF lasting 8.7 seconds. Finally, voltage electrogram mapping was performed in the right and left endocardia and the epicardium (5444 recording sites). No area displayed low-voltage fractionated electrograms, which would have indicated localized structural heart disease.^5^ The patient refused genetic testing. A subcutaneous implantable cardioverter-defibrillator was inserted, and no pharmacologic antiarrhythmic treatment was prescribed. No VF occurred during 14 months of follow-up.

### Case 2

A 40-year-old woman with no family history of sudden cardiac death was admitted after a VF episode that had been successfully defibrillated. The event occurred while the patient was at rest during a party, without any drug or alcohol consumption.

During hospitalization, the patient’s ECG showed sinus rhythm with QRS duration of 88 ms and no ectopy (Figure 1D). Cardiac evaluation including magnetic resonance imaging and pharmacologic tests demonstrated no structural heart disease or known electrical syndrome.

**Figure 3:**
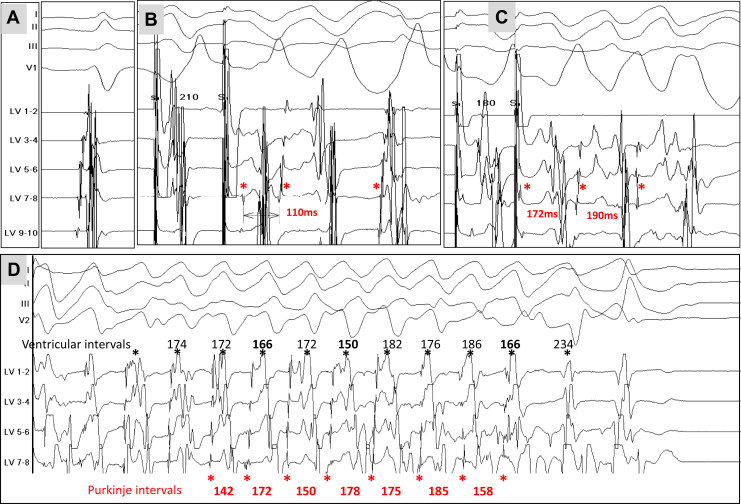
Patient 2. **A:** Recording during sinus rhythm at multiple sites on the distal left posterior fascicle. Purkinje potentials are recorded immediately before the onset of the ventricular complex. The same catheter position is kept during programmed stimulation (panel B). **B:** Extrastimulation (210 ms, drive cycle 600 ms) from the distal Purkinje. Note that the 2 initial Purkinje activations (cycle 110 ms) are different, indicating involvement of a different part of peripheral Purkinje system. **C:** Extrastimulation at a coupling interval of 180 ms. There is no latency after pacing (no delay stimulus–Purkinje) compared to extrastimulus at 210 ms. A polymorphic ventricular tachycardia is shown in panel **D**. The changes in ventricular cycles (*asterisks* and values in *black*) were constantly preceded by a similar change in Purkinje cycles (*asterisks* and values in *red*). A shortening in Purkinje cycles (142,150,145 ms) immediately precedes the shortening in ventricular cycles (166, 150, 166 ms). Note that arrhythmia termination is preceded by cessation of Purkinje activity.

Electrophysiological study was performed with the same protocol as used in case 1. Voltage mapping from the endocardium and epicardium (3067 sites) did not detect any abnormal areas. The HV interval was 45 ms. Programmed ventricular stimulation was unable to induce VF from the right ventricle. The decapolar catheter was positioned along the left Purkinje posterior fascicle, as described earlier. Although no abnormality was noted using a single extrastimulus, double extrastimuli using S2 at 270 ms and S3 below 230 ms induced rapid responses in the distal Purkinje system (Figure 3). They were consistently induced with variations in Purkinje intervals and activation sequence. Four episodes of polymorphic VT were provoked with a maximal duration of 4.2 seconds. During polymorphic VT, the variations in ventricular cycles were constantly preceded by a similar change in Purkinje cycles. Figure 3D shows the shortening of Purkinje cycles preceding the shortening of ventricular cycles. A subcutaneous implantable cardioverter-defibrillator was inserted, and no pharmacologic antiarrhythmic treatment was prescribed. No VF occurred during 9 months of follow-up. Results of wide-arrhythmia (31-gene) genetic testing were negative.

## Discussion

We report here the cases of 2 patients with idiopathic VF in whom electrophysiological observations indicated abnormal properties in the peripheral Purkinje network without identification of any other cause of VF.

The patients presented with sentinel cardiac arrest due to VF. Rare fascicular ectopy with a long coupling interval was documented after the event in 1 patient; no ectopy was noted in the other patient. Short-coupled R-on-T ectopy initiating idiopathic VF and originating from the distal Purkinje system has been described in previous series.^1^, ^2^ The most commonly reported mechanisms are triggered activity and abnormal automaticity.^8^, ^9^, ^10^

Despite the absence of clinically significant ectopy and the normal QRS complexes, the electrophysiological investigations provided important insights into a pathogenic role of the Purkinje system. First, VF that was not inducible from the right ventricular apex could be provoked by pacing close to the distal left Purkinje system. Second, electrophysiological abnormalities—short cycle lengths and complex conduction sequences—were observed in parts of the distal Purkinje system despite a normal HV interval at baseline. Cycle lengths <200 ms were observed well below the normal refractory periods. Long conduction times, fragmented Purkinje activity, and dissociated activity between contiguous bipoles also were suggestive of alterations in the Purkinje system or its myocardial connections. The Purkinje system, which couples to the myocardium at discrete Purkinje–myocardial junctions, displays asymmetrical conduction properties at these interfaces with respect to the anterograde or retrograde direction.^8^, ^9^ Although loading effects (source–sink mismatch) favor retrograde conduction, the long Purkinje refractory period normally prevents retrograde propagation at short coupling intervals. In our patients, the pacing protocol performed near the distal junctions nevertheless resulted in activation invading the Purkinje system retrogradely from the myocardium. We were unable to track the conduction pathway and precisely characterize Purkinje properties, but recently developed wide-field catheters may be helpful for this purpose in the future. A structural or electrical pathology may be responsible for the heterogeneity of refractoriness in Purkinje fibers, leading to nonuniform conduction across the strands (defective gate function).^8^ If Purkinje involvement is spatially limited, it may constitute a target for ablation.

The induction of repetitive activity within the peripheral Purkinje system was the most striking finding in our patients. This was confirmed by the reproducibility of induction in both patients and the consistency of distal Purkinje potentials, which preceded changes in ventricular activation. The mechanism cannot be specified in the patients but could be triggered activity and/or reentry. The polymorphic QRS morphology was suggestive of a gradual shift in trajectory and ventricular exit from the Purkinje network, which has been demonstrated in computer modeling studies.^10^, ^11^ The critical conditions of reentry dynamics in relation to the heterogeneity of Purkinje–myocardial junctions were shown by Gilmour and Watanabe^12^ in various experiments of canine preparations. In addition, Lazzara et al^13^ showed that the (proximal) bundle branches were the preferential sites for conduction block during premature stimulation, whereas conduction could still occur through some distal fibers (“*interfascicular fibers of the left bundle branch and septal fibers of the right bundle branch”*). In computer modeling, Deo et al^11^ showed that development of reentry was dependent both on the distal site of excitation (from the distal network) as opposed to proximal fascicle and on impaired conduction in the fascicular system to produce sufficient delay for reentry. Because a conduction disease should be present in the His-bundle branches in order to obtain bundle branch reentry, it is likely that a similar or greater conduction impairment should be present for distal fascicular reentry.

The present case reports demonstrate that some idiopathic VF can be associated with inducible repetitive activity in the peripheral Purkinje system, whether or not ectopy is still present. We have considered these responses to be an abnormal phenomenon because they were not observed in a unique study evaluating left ventricular pacing in control patients.^14^ However, because polymorphic VT may be induced in up to 38% of healthy subjects using double extrastimulation in the right ventricle,^15^ further studies are needed to demonstrate whether some of these “nonspecific” ventricular responses may be located in the Purkinje network and to evaluate their clinical relevance. Challenging the Purkinje conduction retrogradely by pacing close to the Purkinje network is a simple technique as opposed to challenging it anterogradely by His-bundle or fascicular pacing. It may be a means to reveal functional abnormalities in the conduction system that can underlie an arrhythmogenic substrate. With further studies in normal and arrhythmic populations and refinement in the mapping technique, this approach may offer a method for detecting a “Purkinjopathy” in patients with subclinical conduction disturbances who are at risk for ventricular arrhythmias and sudden cardiac death.

## Conclusion

We reported 2 patients with unexplained sudden cardiac death due to VF, which was associated with electrical alterations and inducible repetitive activity in the peripheral Purkinje system.
